# From Gold to Polymer: Latex Bead-Assisted CRISPR-Cas12a Platform for Next-Generation Protein Diagnostics

**DOI:** 10.1021/acsmeasuresciau.6c00030

**Published:** 2026-03-30

**Authors:** Rana Jahani, Jun Chen, Juanhua Kong, Shuo Zhou, Haiyan Zheng, Sathishkumar Munusamy, Xiyun Guan

**Affiliations:** Department of Chemistry, 14716 University of Missouri, Columbia, Missouri 65211, United States

**Keywords:** latex beads, IL-6, CRISPR-Cas12a, fluorescence, barcoding DNA

## Abstract

Sensitive and accurate detection of protein biomarkers in clinical samples is crucial for early disease diagnosis and monitoring treatment outcomes. Although gold nanoparticle (AuNP)-assisted CRISPR-Cas12a biosensors have shown promise, their practical applications have been restricted by limitations of AuNPs, such as costly and complex synthesis, tendency to aggregate, and nonspecific adsorption of various unwanted species. To address these limitations, herein, we report a CRISPR-Cas12a-based fluorescence biosensor for the detection of a well-established and clinically significant inflammatory biomarker, interleukin-6 (IL-6), which employs latex beads instead of AuNPs as a transducer and signal amplifier. By the combined use of IL-6 detection antibody-functionalized magnetic beads, dual-functionalized latex beads, which carry both IL-6 detection antibodies and multiple dsDNA strands per antibody molecule, and the Cas12a-crRNA system, our method was able to detect IL-6 with a limit of detection (LOD) reaching as low as 0.76 pg/mL. Moreover, our sensor was highly selective: nontarget proteins such as human serum albumin (HSA), bovine serum albumin (BSA), C-reactive protein (CRP), procalcitonin (PCT), and IL-2β would not interfere with the detection of IL-6. In addition, the practical application of this sensor was assessed by successfully analyzing simulated serum samples. This study shows that latex beads can serve as an alternative to gold nanoparticles for CRISPR-based protein diagnostics. This approach improves stability and specificity while maintaining a high level of sensitivity for early disease diagnosis.

## Graphical abstract

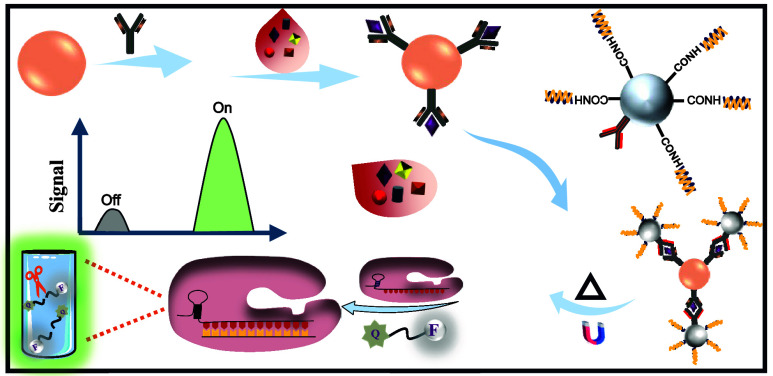

## Introduction

Proteins are central to nearly all physiological processes and can serve as critical biomarkers for disease diagnosis, prognosis, and therapeutic monitoring.^1,2^ Accurate and sensitive detection of proteins at ultralow concentrations is therefore essential for advancing precision medicine, enabling early diagnosis of conditions such as cancer, infectious diseases, and neurodegenerative disorders.^3–6^ Traditional immunoassays, including ELISA,^7–9^ Western blotting,^10,11^ and mass spectrometry,^12,13^ have long been used for protein detection. However, they often lack the required sensitivity, involve lengthy protocols, or depend on expensive and specialized instrumentation.

In recent years, nanomaterial-assisted CRISPR-Cas12a platforms have emerged as powerful tools for protein detection.^14–22^ In particular, gold nanoparticles (AuNPs) have been extensively utilized as signal carriers and amplifiers in immuno-CRISPR assays.^23,15^ Their unique optical and electronic properties, high surface-to-volume ratio, and capacity to be conjugated with antibodies and DNA make them excellent mediators for converting protein binding events into amplified nucleic acid signals. Indeed, AuNP-assisted CRISPR assays have achieved attomolar sensitivity for clinically relevant biomarkers such as neurofilament light chain (NfL),^24^ interleukins,^25^ and proteases.^26^ Despite these successes, AuNPs suffer from inherent drawbacks that limit their broader applicability.^27,28^ They are costly to synthesize, prone to aggregation in complex biological matrices, and susceptible to nonspecific adsorption of proteins and nucleic acids, which can compromise reproducibility and assay stability.^29^ These limitations highlight the need for alternative nanomaterials that retain the amplification advantages of AuNPs while offering greater stability, lower cost, and improved functionalization.

In this work, latex (polystyrene) beads (PS particles or PSPs in short) were introduced instead of AuNPs as the next generation of signal transducers, which may constitute a systematic disruption in the domain of immunoassay design. PSPs are monodisperse polymer particles, typically ranging from nanometers to micrometers, and can be engineered with a wide variety of surface functional groups that allow versatile bioconjugation strategies.^30–33^ They are extensively used as visible labels in immunoassays, where antibodies or other biomolecules can be immobilized either through passive adsorption or, more effectively, via covalent coupling for enhanced stability and performance.^33–35^ The choice of functionalization is critical since the biological activity of the immobilized molecule strongly depends on the chemical and physical environment of the bead surface. By tailoring surface chemistry, latex beads can serve diverse roles including tracers in agglutination tests, solid-phase supports in immunoassays, carriers for targeted delivery in vaccines or gene therapy, and recognition elements in molecular biology. In addition, they are employed in surface modification of flat supports, coatings on other colloids, and as platforms for diagnostic and therapeutic applications, highlighting their broad utility across biotechnology and nanomedicine.^35,36^ Compared with AuNPs, latex beads have several built-in advantages. For example, they are cost-effective, and can be made with very precise sizes and a wide range of surface functional groups.^37–40^ In particular, latex beads are generally more stable and less prone to aggregation than AuNPs due to differences in their surface chemistry, stabilization mechanisms, and interparticle interactions. In our protein sensor design, EDC/NHS chemistry was used to immobilize detection antibodies and multiple DNA strands per antibody molecule to the surface of carboxyl-functionalized PSPs. This strategy enables convenient conversion and amplification of protein-antibody binding events into barcoding DNA with predesigned sequences. As a proof of concept, we selected interleukin-6 (IL-6), a well-established and clinically significant inflammatory biomarker, as the model analyte for demonstrating the feasibility of our platform. The assay workflow starts with an antibody–protein–antibody sandwich made of magnetic beads (MBs). This sandwich only isolates the target protein from the sample, which makes the separation convenient and highly specific. Note that the MB–analyte–PSP complex will only form when the analyte is present, which helps reduce false positives and background noise. When the barcoding DNA on the PSPs is heated in a controlled way, it is released into the solution. The MBs and other parts can be easily removed with a magnetic field. The released DNA then activates CRISPR-Cas12a, with carefully designed guide RNAs ensuring that only the specific released sequences cause collateral cleavage. The fluorescence enhancement driven by the CRISPR system not only makes the signal stronger, but it also transforms the presence of the analyte into direct, quantitative, and qualitative readouts. Taken together, our sensing platform employes cost-effective and stable PSPs as signal transducers, and take advantage of the high specificity of the functionalized magnetic beads and the strong amplification power of CRISPR-Cas12a to perform next-generation protein diagnostics.

## Experimental Section

### Materials and Reagents

Both carboxylated MBs (∼2 × 10^9^ beads/mL) and carboxyl latex beads with sizes from 100 to 500 nm were purchased from Invitrogen (Carlsbad, CA, USA). IL-6 protein and the corresponding monoclonal capture and detection antibodies were obtained from MyBiosource (San Diego, CA, USA). Other chemicals and reagents like HSA, BSA, CRP, PCT, IL-2β, Tween-20, and pooled human serum (AB male plasma origin, USA) were ordered from Sigma-Aldrich (St. Louis, MO, USA). Synthetic crRNA, LbCas12a enzyme, and other oligonucleotides, including dye-labeled ssDNA reporter (sequence: FAM-TTATT-IABkFQ), were purchased from Integrated DNA Technologies (IDT, Coralville, IA, USA).

### Preparation of Functionalized MBs

MBs functionalized with monoclonal capture antibodies (Ab_1_) against IL-6 were prepared according to our previously reported EDC/NHS coupling protocol with minor modifications.^41,42^ Briefly, carboxylated MBs (200 μL) were washed three times with MES buffer (25 mM, pH 5.0) and resuspended in the same buffer. The carboxyl groups were then activated by addition of EDC (100 μL, 50 mg/mL) and NHS (100 μL, 50 mg/mL) under gentle agitation for 30 min at room temperature. After removal of the supernatant and thorough washing, IL-6 capture antibody (100 μL, 1 mg/mL) was introduced and incubated with rotation for 30 min to allow covalent coupling. To minimize nonspecific binding, the antibody-conjugated MBs were blocked with BSA solution (0.05%) for 10 min, washed, and finally resuspended in PBS (pH 7.4). The prepared functionalized MBs (Ab_1_-MBs) were stored at 4 °C until further use.

### Synthesis of Dual-Functionalized Latex Beads

EDC/NHS coupling^43^ was used to prepare functionalized PSPs by incubating carboxyl latex beads with a premix of detection antibody (Ab_2_) and amine functionalized T_20_ DNA (P1, sequence: TTTT­TTTT­TTTT­TTTT­TTTT) with a certain molar ratio. Briefly, 100 μL of carboxylated latex beads (undiluted or after 1:100 dilution) was rinsed three times with 200 μL of MES buffer that contains 1% Tween-20 (25 mM, pH 5.0) and then resuspended in the same buffer. To activate the carboxyl groups, 50 μL of EDC solution (50 mg/mL) and 50 μL of NHS solution (50 mg/mL) were added to the bead suspension. The mixture was agitated at room temperature for 30 min to ensure proper activation. Afterward, the supernatant was removed, and the beads were washed thoroughly by centrifuging three times using 100 μL of fresh MES (containing 1% Tween-20) buffer. Subsequently, the prepared premix (with the total volume ranging from 57.5 to 125 μL, while keeping the DNA concentration and volume constant at 100 μM and 50 μL, respectively) was introduced to the activated latex beads followed by incubation with gentle rotation at room temperature for 30 min to allow effective antibody binding. Then, the latex beads were washed three times with 100 μL of PBS buffer with 0.25% Tween-20 (pH 7.4). To prevent nonspecific binding, the beads were treated with 50 μL of a 0.05% BSA solution for 10 min with occasional vortexing. Finally, the antibody/DNA-conjugated latex beads (Ab_2_-PSP-P1) were resuspended in 100 μL of PBS and stored at 4 °C until further use. Prior to sandwich formation, a complementary DNA strand (A_20_, sequence: AAAA­AAAA­AAAA­AAAA­AAAA) was added to Ab_2_-PSP-P1 to form the final dual-functionalized latex beads product, Ab_2_-PSP-P1-P2. Annealing was achieved through thermal cycling, in which Ab_2_-PSP-P1 (100 μL) and P2 (20 μL, 1 mM) mixture was heated to 70 °C and then gradually cooled to room temperature, ensuring stable duplex formation on the bead surface. Note that, except the “Improving Sensor Sensitivity” section, where 100x diluted carboxylated latex beads were used, undiluted latex beads were employed to prepare functionalized PSPs throughout this entire investigation.

### Procedure for the Latex Bead-Based IL-6 Assay

For IL-6 detection, 50 μL of capture antibody–functionalized magnetic beads (Ab_1_-MBs) were washed three times with 500 μL of assay buffer (10 mM PBS, 0.1 M NaCl, 0.1% BSA, and 0.025% Tween-20, pH 7.2). The beads were resuspended in 1 mL of assay buffer and incubated with IL-6 standard solutions ranging from 2 to 200 pg/mL for 1 h at room temperature with gentle rotation to allow efficient antigen capture. After incubation, the beads were magnetically separated, and the MB-Ab_1_-IL-6 complexes were washed three times with assay buffer to remove unbound proteins. Next, 10 μL of dual-functionalized latex beads (Ab_2_-PSP-P1-P2) was introduced to MB-Ab_1_-IL-6 and incubated for 1 h under slow mixing to form the sandwich structure (MB-Ab_1_-IL-6-Ab_2_-PSP-P1-P2). Unreacted components were removed by magnetic separation, followed by three washes with assay buffer. Then, 100 μL of ultrapure water was added, and the suspension was heated at 70 °C for 15 min. This step selectively released the P2 strand into the solution, while P1 remained associated with the latex beads. The supernatant containing P2 was collected and followed by downstream CRISPR analysis. For the Cas12a trans-cleavage assay, LbCas12a (1 μM) and the corresponding crRNA (2 μM) were preincubated in 1× NEB buffer 3.1 for 30 min at room temperature. The P2-containing supernatant was then added to activate Cas12a for 30 min, followed by addition of a fluorophore–quencher ssDNA reporter (FAM–TTATT–IABkFQ). The mixture was incubated for 3 h at room temperature, and fluorescence was measured at λ_ex/em_ = 492/528 nm to quantify IL-6 levels.

## Results and Discussion

### Principle of Latex Bead-Based IL-6 Detection Platform

The schematic representation of our developed IL-6 assay is illustrated in Figure 1. AuNPs are typically employed in biosensing due to their high surface area and Au–S chemistry, which facilitates signal conversion and amplification. The distinctive feature of this work is the use of latex beads instead of AuNPs as a transducer and signal amplifier for highly sensitive IL-6 detection due to their lower synthesis cost and higher resistance to aggregation than AuNPs. Our pioneering latex beads-based IL-6 biosensing platform consists of three major components: IL-6 capture antibody-functionalized magnetic beads (MB-Ab_1_), dual-functionalized latex beads (Ab_2_-PSP-P1-P2) which carry both IL-6 detection antibodies and multiple dsDNA strands per antibody molecule via EDC/NHS coupling chemistry, and the CRISPR-Cas12a system. Briefly, MB-Ab_1_, the IL-6 containing sample, and Ab_2_-PSP-P1-P2 will be incubated to form sandwich complexes (MB-Ab_1_-IL-6-Ab_2_-PSP-P1-P2). After magnet separation and removing other matrix components, the obtained sandwich complexes will be resuspended in ultrapure water, followed by heat denaturation to dehybridize the dsDNA. The released barcoding ssDNA P2 is collected for downstream CRISPR-Cas12a analysis, where P2, under the guidance of crRNA, activates Cas12a, which then cleaves a dye-labeled reporter ssDNA, resulting in fluorescence emission.

**Figure 1:**
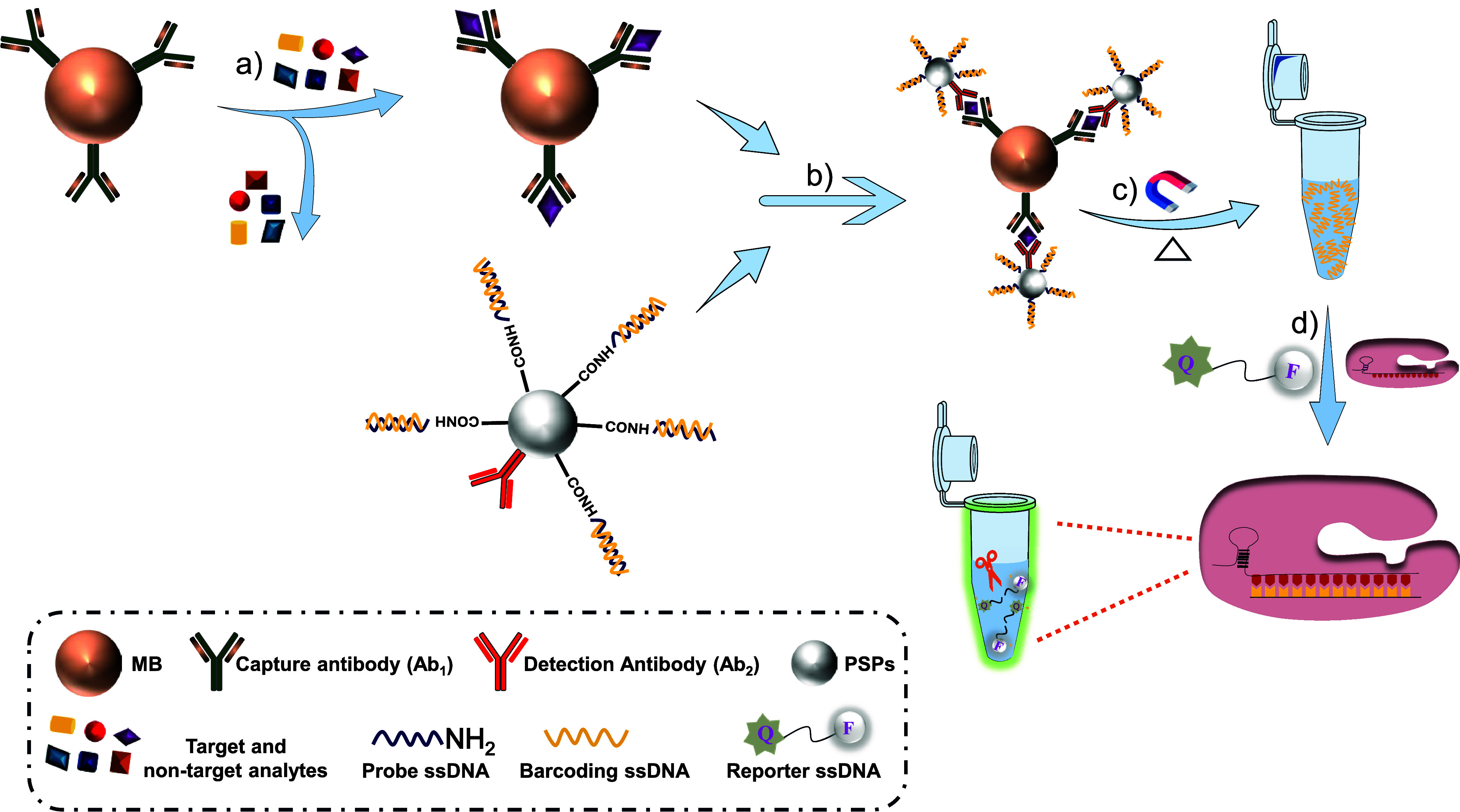
Schematic illustration of the latex bead-assisted CRISPR-Cas12a sensing platform for IL-6 detection (not to scale). (a) In the presence of IL-6, (b) it will form sandwich complex with IL-6 capture antibody-functionalized MBs and dual-functionalized latex beads which carry both the IL-6 detection antibodies and barcoding dsDNA. (c) Controlled heating of the sandwich complex at an appropriate temperature (70–90 °C) leads to the releasing of barcoding ssDNA from the latex beads, (d) which then activates Cas12a–crRNA, triggering collateral cleavage of a fluorophore–quencher ssDNA reporter and thus leading to fluorescence emission.

### Characterization of Functionalized MBs and PSPs

Verifying the successful functionalization of MBs and latex beads is crucial to ensuring sensor functionality. For this purpose, zeta potential measurements of MBs were first carried out. We found out that, before Ab_1_ modification, MBs showed a zeta potential value of −30.6 mV, indicating a pronounced negative surface charge, due to the abundance of carboxyl groups on MBs. In contrast, the modified MBs had a zeta potential of −28.1 mV, providing evidence for the successful immobilization of Ab_1_ to MBs. The reason why the zeta potential became less negative is because of the decreasing number of negative carboxyl groups available to contribute to the zeta potential after covalent coupling of the amine groups on the antibodies to the carboxyl groups on the beads (Figure 2A). To quantitatively evaluate the extent of antibody conjugation, the UV–vis absorbance (at 280 nm) of the antibody solution before and after the coupling reaction was monitored. Based on an initial antibody input of 50 μg and approximately 26.9 μg of IL-6 capture antibody being immobilized onto the MBs (Supporting Information, Figure S1), the coupling efficiency was determined to be 53.8%, and the average antibody surface density was estimated to be ∼5.4 × 10^5^ antibodies per MB. This high antibody loading density is consistent with efficient carbodiimide coupling on carboxylated magnetic beads and provides a quantitative basis for the strong target capture capability of the Ab_1_-functionalized MBs. Furthermore, the preserved sensing performance observed in subsequent IL-6 detection experiments confirms that the immobilized antibodies retain sufficient bioactivity following covalent conjugation. Collectively, these results demonstrate that the EDC/NHS coupling strategy yields MBs with a high density of functionally active capture antibodies, making them well-suited for sensitive and reliable IL-6 detection.

**Figure 2:**
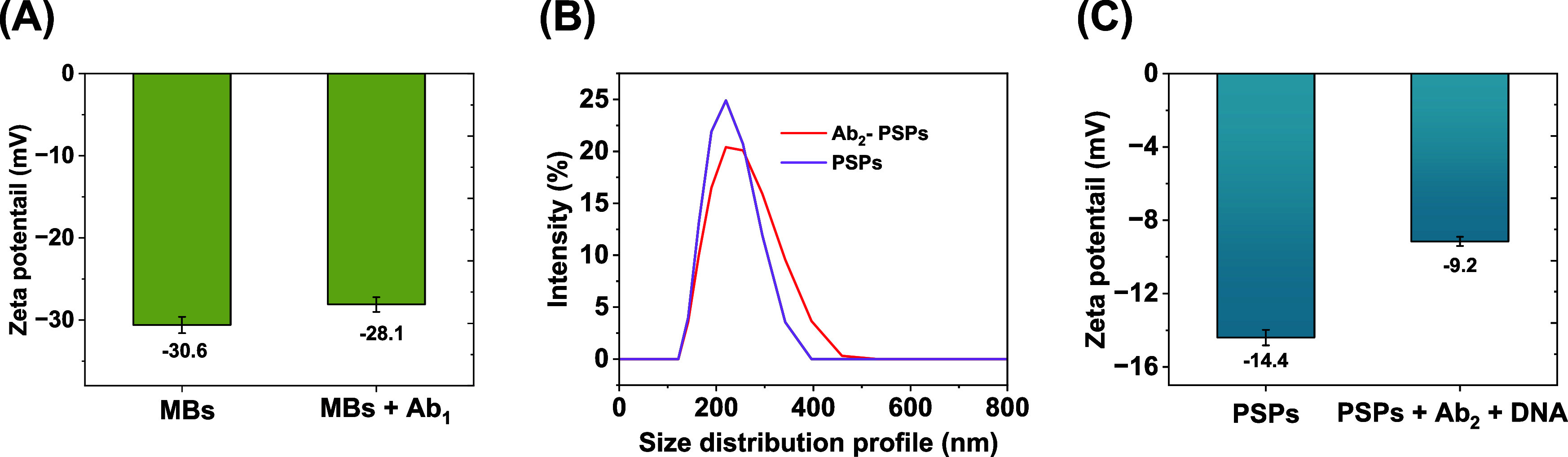
Characterization of functionalized MBs and latex beads. (A) Surface zeta potential of the free MBs and Ab_1_-functionalized MBs, (B) hydrodynamic size distribution of bare latex beads and dual-functionalized latex beads with Ab_2_ and DNA, and (C) surface zeta potential of unmodified latex beads and dual-functionalized latex beads with Ab_2_ and DNA. In Figure 2A,C, all the samples were resuspended in HPLC grade water before zeta potential measurement.

As to dual-functionalized PSPs, dynamic light scattering (DLS) and zeta potential analyses were used to validate the concurrent modification of 200 nm latex beads with Ab_2_ and DNA through EDC/NHS coupling, with the results summarized in Figure 2B,C. Briefly, consistent with the presence of an extra protein layer and related hydration effects, the DLS measurements showed a noticeable increase in the bead size from 220 nm in the unmodified state to 231 nm after antibody attachment. The successful bioconjugation was further confirmed by the surface charge analysis. Because of the carboxyl groups, bare latex beads showed a strong negative potential of −14.4 mV (Figure 2C). This negativity was significantly decreased to −9.2 mV upon conjugation with the IL-6 detection antibody and DNA, indicating surface coverage by the protein layer and DNA. Furthermore, FT-IR spectrum (Figure S2) of free carboxylated latex beads exhibits a weak but discernible band at ∼1696 cm^–1^, assigned to the C=O stretching vibration of surface carboxylic acid (−COOH) groups, whose low intensity is expected due to the limited surface coverage and the surface-sensitive nature of ATR-FTIR.^44^ Following EDC/NHS-mediated immobilization of double-stranded DNA, this carbonyl band is markedly attenuated, consistent with the consumption of −COOH groups and their conversion into amide (−CONH−) linkages. Owing to the low grafting density and strong polymer background, the amide vibrations do not appear as distinct peaks but contribute to broader overlapping features. Concurrently, the aromatic C=C stretching band of the polystyrene matrix near ∼1600 cm^–1^ becomes broadened after DNA functionalization, attributable to the superposition of nucleobase ring vibrations and possible amide-related modes, indicating the formation of a biomolecule-modified surface. The characteristic polystyrene fingerprint band at ∼1030 cm^–1^ is significantly weakened, reflecting surface masking by the DNA layer and reduced contribution from the underlying polymer. Importantly, the DNA-functionalized beads display enhanced absorption in the 1200–1100 cm^–1^ region, characteristic of sugar–phosphate backbone vibrations (P–O and C–O stretching) of DNA, providing direct spectroscopic evidence of successful DNA immobilization. In addition, UV–Vis absorption spectrum (Figure S3) of the free latex beads displays a strong absorption maximum at ∼225 nm, characteristic of π–π* transitions of the aromatic phenyl rings in the polystyrene matrix. Upon functionalization with double-stranded DNA, this absorption maximum shifts bathochromically to ∼230 nm with a slight change in overall absorbance intensity. This red shift indicates a modification of the local electronic environment at the bead surface, consistent with successful DNA attachment and altered surface chemistry. Taken together, the combined surface charge modulation and increase in hydrodynamic size offer a strong evidence for the effective dual-functionalization of latex beads with DNA and antibody, thus creating a stable nanoconjugate platform for downstream biosensing.

### Detection of IL-6

As a proof of concept, initial experiments were carried out to examine a series of IL-6 proteins with varying concentrations ranging from 2 to 200 pg/mL by using the dual-functionalized latex beads prepared with an Ab_2_/DNA premix molar ratio of 1:10 and 200 nm diameter bead size, and functionalized MBs as well as the CRISPR-Cas12a system according to the procedure described in the Experimental Section. Our experimental results showed that the fluorescence signals of the IL-6 samples increased steadily as their concentrations increased (Figure 3A). The limit of detection (LOD) at a 99.7% confidence level for IL-6 was determined to be 0.76 pg/mL using the formula LOD = 3σ/*S* ^45,46^ and based on a linear regression (*y* = 31.69*x* + 1153, and *R* ^2^ = 0.9973) after fitting the dose–response curve (Figure 3B), where σ is the standard deviation of the blank signal and S is the slope of the calibration curve. To the best of our knowledge, such a LOD surpasses various most sensitive IL-6 assays reported to date except the one we developed recently using the AuNP-assisted CRISPR assay,^24^ which typically detect IL-6 in the range of several pg/mL to ng/mL levels (Supporting Information, Table S1). Note that, in healthy individuals, blood IL-6 levels are low (around 5 pg/mL), but they can rise significantly during inflammation, infection, or other diseases to between 100 pg/mL and 1 ng/mL. Therefore, our developed proof-of-concept latex beads-assisted IL-6 sensing platform is sensitive enough to be utilized for analysis of clinical samples. The exceptional analytical performance of our platform, including its broad dynamic range and ultralow LOD, is attributed to the dual amplification mechanism: immunocomplex-driven release of barcoding DNA and the collateral cleavage activity of the Cas12a–crRNA complex. Moreover, the use of MBs for target protein enrichment provided high specificity and effectively reduced nonspecific background signals.

**Figure 3:**
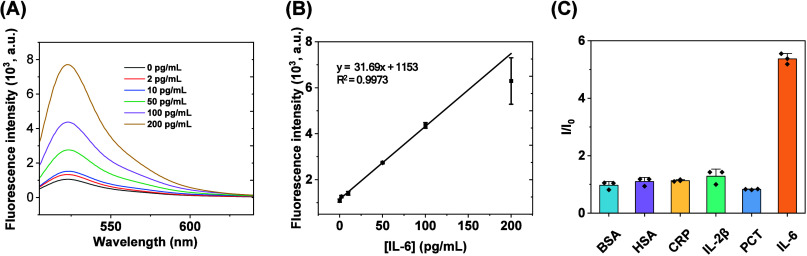
Performance of the latex bead-assisted CRISPR-Cas12a biosensor for IL-6 detection. (A) Fluorescence spectra, showing the concentration-dependent response of the biosensor toward IL-6. (B) Plot of the fluorescence intensity as a function of IL-6 concentration. (C) Selectivity study. The fluorescence intensity values shown in Figure 3B were derived from the fluorescence spectra at 528 nm of Figure 3A. In Figure 3C, except IL-6 (at 200 pg/mL), the concentrations of all the other proteins used were 2000 pg/mL each (*n* = 3).

To evaluate the selectivity of the developed CRISPR-Cas12a-based IL-6 biosensor, a panel of potentially interfering proteins was investigated, including PCT, CRP, IL-2β, HSA, and BSA. Among them, PCT, CRP, and IL-2β are clinically relevant inflammatory biomarkers, while HSA and BSA represent the most abundant serum proteins in humans and bovines, respectively. Each interferent was tested at concentrations 10-fold higher than that of IL-6, with the results summarized in Figure 3C. As expected, negligible fluorescence signals were observed for all nontarget proteins, comparable to those of the negative control, whereas IL-6 produced a significantly elevated response. The results clearly demonstrate that the developed biosensor possesses excellent specificity for IL-6, even in the presence of structurally and functionally related proteins.

### Serum Sample Analysis

The matrix effect is a significant problem in biomarker analysis from biological fluids like blood or urine because the abundance of other compounds can interfere with the detection of low-concentration target analytes. Since undiluted serum produced unreliable results due to the matrix effects, diluted serum samples were investigated in this proof-of-concept demonstration of the potential application of our developed IL-6 sensor in clinical sample analysis (note that serum dilution is a well-established strategy to overcome the matrix effect by reducing the concentration of interfering components from the sample, thus minimizing signal suppression and improving the accuracy of the results). We found that, no IL-6 was detected in a commercial healthy human serum sample (male AB plasma, USA origin; Sigma-Aldrich, St. Louis, MO) after diluted 50 times in PBS, consistent with the fact that the blood IL-6 levels in healthy individuals are low. To assess the sensor performance, two simulated serum samples were analyzed, where known concentrations of IL-6 (2 and 10 pg/mL) were spiked into the diluted serum, with the results summarized in Table 1. Clearly, our developed IL-6 sensor could accurately detect the IL-6 concentrations in these samples, with the IL-6 recoveries obtained ranging from 102 ± 6 to 103 ± 2% (*n* = 3), suggesting that the serum matrix components would not significantly affect the performance of our developed IL-6 biosensor.

**Table 1:** Analysis of Serum Samples with the Developed Latex Bead–Assisted CRISPR-Cas12a IL-6 Biosensor

sample	IL-6 found^a^ (pg/mL)	recovery (100%)	RSD (%)
serum			
serum +2 pg/mL IL-6	2.06 ± 0.04	103	2
serum +10 pg/mL IL-6	10.3 ± 0.6	102	6

a Each experimental value represents the mean of three replicate analyses ± one standard deviation.

### Improving Sensor Sensitivity

The dual-functionalized latex beads significantly influence the overall performance of the sensing platform. In general, as the DNA-to-antibody molar ratio on the bead surface increases, the number of DNA strands per antibody-IL-6 binding event available for downstream CRISPR analysis increases, theoretically enhancing the sensor sensitivity. To systematically assess this effect, we prepared a series of dual-functionalized PSPs (Ab_2_-PSP-P1) by incubating carboxyl latex beads of 200 nm diameter with mixtures of IL-6 detection antibody and amine-functionalized P1 DNA with molar ratios ranging from 1:10 to 1:50 via EDC/NHS coupling. After hybridization with P2, these dual-functionalized PSPs (Ab_2_-PSP-P1-P2), functionalized MBs and the CRISPR-Cas12a system were utilized to detect 50 pg/mL of IL-6. As shown in Figure 4A, with an increasing DNA-to-antibody molar ratio in the reaction mixture, the fluorescence signal-to-noise ratio first increased and then decreased, which was partly in agreement with our prediction. One likely interpretation is that at 1:50 molar ratio of Ab_2_ to DNA, there was inadequate antibody loading on the surface of latex beads, leading to reduced antigen-capture efficiency. One possible solution to this insufficient antibody loading issue is to fine-tune the amount of latex beads used to incubate with Ab_2_ and DNA, which is currently under way in our laboratory.

**Figure 4:**
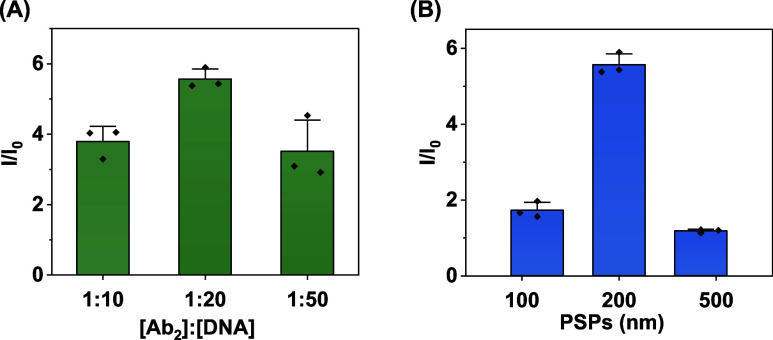
Effect of (A) the molar ratio of Ab_2_ to DNA (P1) in the solution mixture used for preparing the dual-functionalized PSPs and (B) the size of latex bead on IL-6 detection. The concentrations of IL-6 used were 50 pg/mL each.

The size of the latex beads also plays a decisive role in the overall sensitivity of the sensing platform. In principle, the larger the bead size, the more barcoding DNA molecules could be potentially immobilized on the surface of the beads, leading to an increasing molar ratio of barcoding DNA to antibody and hence an enhanced sensor sensitivity. To evaluate the impact of PSP size on IL-6 detection, a series of dual-functionalized PSPs with bead sizes ranging from 100 to 500 nm in diameter was prepared. These PSPs and functionalized MBs were used to detect 50 pg/mL of IL-6. We found that, among them, the latex beads of 200 nm diameter consistently provided the highest sensor sensitivity, while both smaller (100 nm) and larger (500 nm) beads exhibited suboptimal performance (Figure 4B). The worsen performance of the 100 nm-diameter beads can be attributed to their limited surface area, which restricts the number of DNA molecules that could be immobilized, thereby lowering the signal amplification capacity. In contrast, the 500 nm diameter beads, despite having larger surface areas, are more prone to aggregation, thus hampering the colloidal stability and ultimately diminishing the sensitivity of the assay. Taken together, the combined experimental results suggest that regulating the molar ratio of the antibody to DNA immobilized on the latex beads and the size of PSPs offer the potential to improve the sensitivity and LOD of the latex beads-assisted protein assays.

## Conclusions

In summary, by using dual-functionalized latex beads as the signal transducing element, a CRISPR-Cas12a-based fluorescence biosensor was successfully developed for effective and selective detection of IL-6. Compared with the popular AuNP-based systems, the latex bead platform was more stable, cost-effective, and had a wider range of surface chemistries available for coimmobilizing antibodies and DNA. In our sensor design, latex beads played two important roles. First, they served as carriers to convert antibody–IL-6 binding events into barcoding DNA. Second, by regulating the molar ratio of the barcoding DNA to detection antibodies on the latex beads, signal multiplication could be conveniently accomplished, without involving extra time-consuming chemical or enzymatic amplification steps. As a result, our developed latex beads-based assay showed strong analytical performance, as demonstrated by its high sensitivity, wide dynamic range, and great specificity against other inflammatory proteins. Furthermore, simulated serum sample analysis was successfully achieved. It should be noted that the latex beads-based biosensor reported in this work was proof of concept. Better sensor performance and sensitivity will be expected if further optimization of parameters (e.g., optimizing the design of barcode DNA sequences, crRNA efficiency, and the density of antibodies on latex beads) is performed. Moreover, instead of using carboxyl latex beads, other functionalized latex beads may offer the potential for improving signal multiplication. In addition, in this work, the dual-functionalized latex beads were prepared by incubation them with a mixture of barcoding DNA and IL-6 detection antibody in a certain molar ratio. Better regulation of the relative loading density of DNA to antibody on the surface of latex beads might be accomplished by sequential immobilization. Although this entire investigation focused on IL-6, it can be visualized that the sensing strategy developed in this work may be utilized to develop sensors for a wide range of clinically important protein biomarkers, making it possible to develop multiplexed systems for early diagnosis of various diseases. Taken together, this work demonstrates that latex beads can serve as an alternative to gold nanoparticles for CRISPR-based protein diagnostics and opens the avenue to the development of next generation of low-cost platforms for biosensing applications.

## Supplementary Material



## Notes

Additional table and figures, including comparison of LOD in IL-6 detection between different techniques, UV–vis spectra of MBs before and after antibody conjugation, FTIR spectra of PSPs, DNA P1, and Ab_2_-PSP-P1-P2, and UV–vis spectra of PSPs before and after antibody and DNA conjugation (PDF)
