# Supplementary material for: From Gold to Polymer: Latex Bead-Assisted CRISPR-Cas12a Platform for Next-Generation Protein Diagnostics
Source: ACS Meas Sci Au. 2026 Mar 30;6(3):728–35. doi: 10.1021/acsmeasuresciau.6c00030 (PMC13281178; doi:10.1021/acsmeasuresciau.6c00030)
Supplement: Supplementary file 1 [file tg6c00030_si_001.pdf]

# Supporting Information

## From Gold to Polymer: Latex Bead–Assisted CRISPR-Cas12a Platform for Next-Generation Protein Diagnostics

Rana Jahani, Jun Chen, Juanhua Kong, Shuo Zhou, Haiyan Zheng, Sathishkumar Munusamy*, and Xiyun Guan*

**Author affiliation:**

Department of Chemistry, University of Missouri, Columbia, MO 65211, USA

*Corresponding Author e-mails: smvm8@missouri.edu and xgpc2@missouri.edu

### Table of Contents:

**Figure S1.** UV-Vis spectra of MBs before and after antibody conjugation……………….. S-2

**Figure S2.** FTIR spectra of PSPs, DNA P1, and $Ab_2$-PSP-P1-P2…………………..…...… S-3

**Figure S3.** UV-Vis spectra of PSPs before and after antibody and DNA conjugation…..… S-4

**Table S1**. Comparison of LOD in IL-6 detection between different techniques…………...S-5

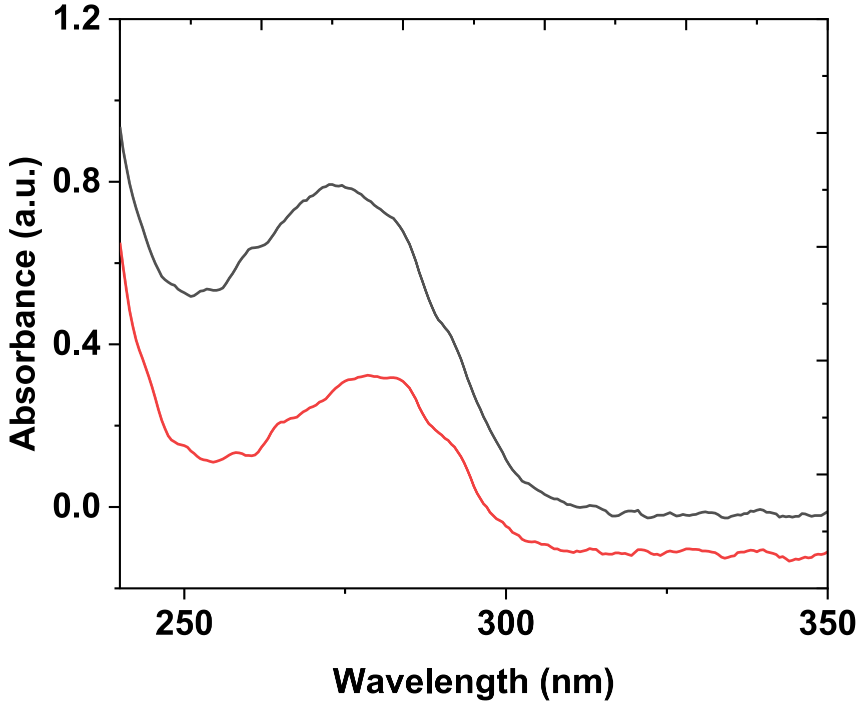


**Figure S1**. UV-Vis absorbance spectra of antibody solution before (shown in black) and after (shown in red) MBs conjugation. The concentration of IL-6 antibody used before modification was 1 mg/mL.

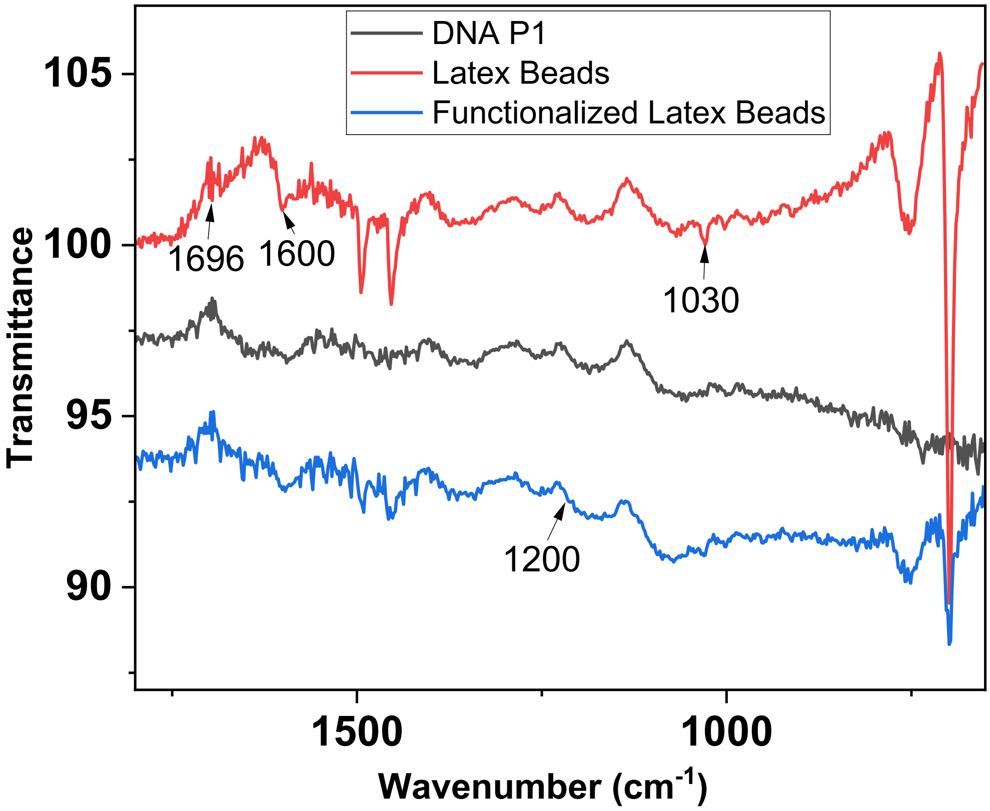


**Figure S2.** FTIR spectra of free latex polystyrene beads (PSPs), DNA P1, and functionalized PSPs ($Ab_2$-PSP-P1-P2). The spectra were collected without applying corrections to the spectra from 32 transmissions scan with 2 $cm^{-1}$ resolution.

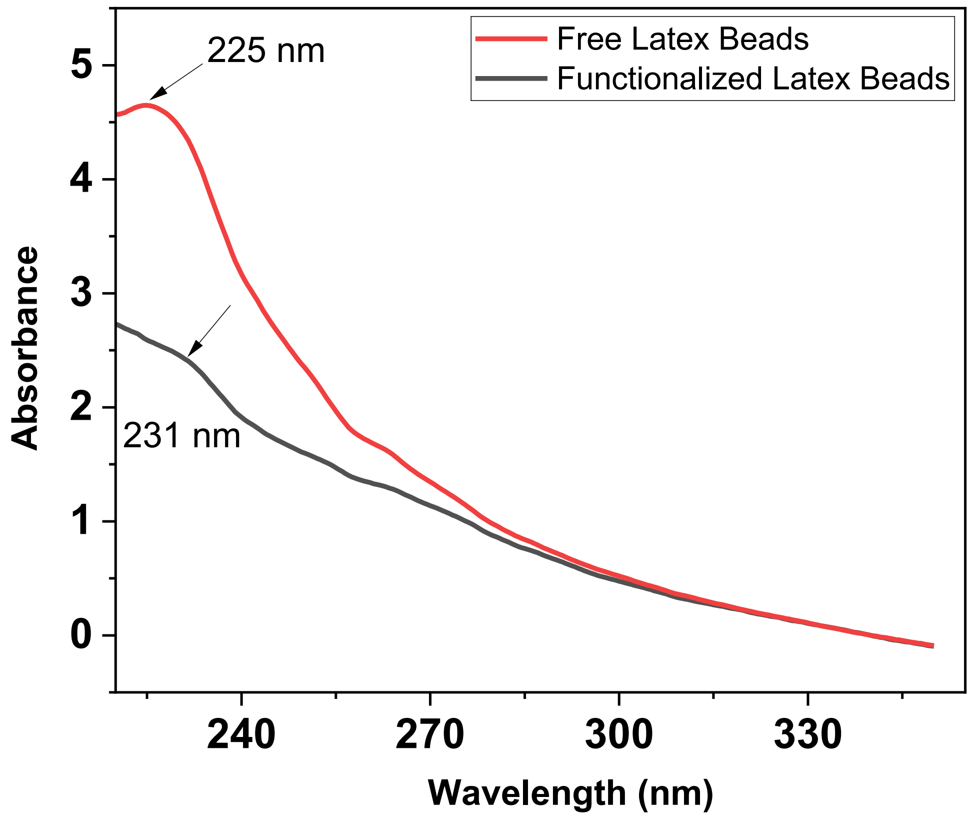


**Figure S3.** UV-Vis spectra of PSPs before (red) and after (black) antibody and DNA conjugation. 37.5 μL of 6.6 μM IL-6 antibody and 50 μL of 100 μM DNA P1 were used for surface modification.

**Table S1**. Comparison of LOD in IL-6 Detection Between Different Techniques

| Materials | Technique | LOD (pg/mL) | Reference |
|---|---|---|---|
| Gold nanorod (AuNR) | ADPAs - LSPRi immunoassay | 4.6 | [1] |
| Selenium nanoparticles (SeNPs) | Lateral flow device (LFDs) | 100 | [2] |
| Quantum dot nanobead | Plasmon-enhanced quantum dot nanobeads (PEQNBs) | 13.6 | [3] |
| AuNPs | Cytokine assay based on optical fiber | 1 | [4] |
| tertButyl 11-azatricyclo [6.2.1.02,7] undeca-2,4,6,9-tetraene-11carboxylate | Fast affinity induced reaction sensor based on a fluorogenic click reaction (FAIRS) | 29 | [5] |
| — | A mass-tagging approach | 19000 | [6] |
| rGO-Ti3C2Tx MXene nanocomposites | Label-free electrochemical assay | 2.1 | [7] |
| AuNPs | Peptide-mediated controllable immunoassay | 12.5 | [8] |
| Single-walled carbon nanotube | Optical nanosensor | 25 | [9] |
| Graphene nanocomposite | Electrochemical biosensor | 2.6 | [10] |

**References**

[1] He, J.; Zhou, L.; Huang, G.; Shen, J.; Chen, W.; Wang, C.; Kim, A.; Zhang, Z.; Cheng, W.; Dai, S.; Ding, F.; Chen, P. Enhanced Label-Free Nanoplasmonic Cytokine Detection in SARS-CoV-2 Induced Inflammation Using Rationally Designed Peptide Aptamer. *ACS Appl. Mater. Interfaces* **2022**, *14* (43), 48464–48475. https://doi.org/10.1021/acsami.2c14748.

[2] Bradley, Z.; Coleman, P. A.; Courtney, M. A.; Fishlock, S.; McGrath, J.; Uniacke-Lowe, T.; Bhalla, N.; McLaughlin, J. A.; Hogan, J.; Hanrahan, J. P.; Yan, K.-T.; McKee, P. Effect of Selenium Nanoparticle Size on IL-6 Detection Sensitivity in a Lateral Flow Device. *ACS Omega* **2023**, *8* (9), 8407–8414. https://doi.org/10.1021/acsomega.2c07297.

[3] Li, X.; Yan, W.; Wang, Y.; Yu, R.; Han, H.; Zhang, P. Plasmon-Enhanced Quantum Dot Nanobead-Based Lateral Flow Assay with Lower Background and Improved Sensitivity. *ACS Appl. Nano Mater.* **2025**, *8* (8), 4120–4128. https://doi.org/10.1021/acsanm.4c07090.

[4] Liu, G.; Zhang, K.; Nadort, A.; Hutchinson, M. R.; Goldys, E. M. Sensitive Cytokine Assay Based on Optical Fiber Allowing Localized and Spatially Resolved Detection of Interleukin-6. *ACS Sens.* **2017**, *2* (2), 218–226. https://doi.org/10.1021/acssensors.6b00619.

[5] Liu, J.; Abdullah, M. A. A.; Yang, L.; Wang, J. Fast Affinity Induced Reaction Sensor Based on a Fluorogenic Click Reaction for Quick Detection of Protein Biomarkers. *Anal. Chem.* **2020**, *92* (1), 647–653. https://doi.org/10.1021/acs.analchem.9b04502.
[6] Ahn, S.; Freedman, D. S.; Massari, P.; Cabodi, M.; Ünlü, M. S. A Mass-Tagging Approach for Enhanced Sensitivity of Dynamic Cytokine Detection Using a Label-Free Biosensor. *Langmuir* **2013**, *29* (17), 5369–5376. https://doi.org/10.1021/la400982h.
[7] Gupta, R.; Kalkal, A.; Mandal, P.; Paital, D.; Brealey, D.; Tiwari, M. K. Label-Free Electrochemical Interleukin-6 Sensor Exploiting rGO-Ti3C2Tx MXene Nanocomposites. *ACS Appl. Mater. Interfaces* **2025**, *17* (31), 44112–44122. https://doi.org/10.1021/acsami.5c06701.
[8] Ran, B.; Zheng, W.; Dong, M.; Xianyu, Y.; Chen, Y.; Wu, J.; Qian, Z.; Jiang, X. Peptide-Mediated Controllable Cross-Linking of Gold Nanoparticles for Immunoassays with Tunable Detection Range. *Anal. Chem.* **2018**, *90* (13), 8234–8240. https://doi.org/10.1021/acs.analchem.8b01760.
[9] Gaikwad, P.; Rahman, N.; Parikh, R.; Crespo, J.; Cohen, Z.; Williams, R. M. Optical Nanosensor Passivation Enables Highly Sensitive Detection of the Inflammatory Cytokine Interleukin-6. *ACS Appl Mater Interfaces* **2024**, *16* (21), 27102–27113. https://doi.org/10.1021/acsami.4c02711.
[10] Ghosh, D.; Tabassum, R.; Sarkar, P. P.; Rahman, M. A.; Jalal, A. H.; Islam, N.; Ashraf, A. Graphene Nanocomposite Ink Coated Laser Transformed Flexible Electrodes for Selective Dopamine Detection and Immunosensing. *ACS Appl. Bio Mater.* **2024**, *7* (5), 3143–3153. https://doi.org/10.1021/acsabm.4c00166.
